# Personal socio‐cultural preferences modulate neural correlates of decisions to socialize with powerful persons

**DOI:** 10.1002/hbm.25963

**Published:** 2022-06-04

**Authors:** Jui‐Hong Chien, I‐Tzu Hung, Joshua Oon Soo Goh, Li‐Wei Kuo, Wei‐Wen Chang

**Affiliations:** ^1^ Graduate Institute of International Human Resource Development National Taiwan Normal University Taipei Taiwan; ^2^ Department of Psychological and Brain Sciences Boston University Boston Massachusetts USA; ^3^ Graduate Institute of Brain and Mind Sciences National Taiwan University Taipei Taiwan; ^4^ Department of Psychology National Taiwan University Taipei Taiwan; ^5^ Neurobiology and Cognitive Science Center National Taiwan University Taipei Taiwan; ^6^ Center for Artificial Intelligence and Advanced Robotics National Taiwan University Taipei Taiwan; ^7^ Institute of Biomedical Engineering and Nanomedicine National Health Research Institutes Miaoli Taiwan; ^8^ Institute of Medical Device and Imaging National Taiwan University College of Medicine Taipei Taiwan

**Keywords:** cultural intelligence, fMRI, power distance, social power, uncertainty avoidance

## Abstract

Social power differences fundamentally shape the behavioral interaction dynamics of groups and societies. While it has long been recognized that individual socio‐cultural preferences mitigate social interactions involving persons of power, there is limited empirical data on the underlying neural correlates. To bridge this gap, we asked university student participants to decide whether they were willing to engage in social activities involving their teachers (higher power status), classmates (equal power status), or themselves (control) while functional brain images were acquired. Questionnaires assessed participants' preferences for power distance, uncertainty avoidance, and cultural intelligence. As expected, participants generally accepted more social interactions with classmates than teachers. Also, left inferior frontal activity was higher when accepting than when rejecting social interactions with teachers. Critically, power distance preferences further modulated right lateral frontoparietal activity contrasting approach relative to avoidance decisions towards teachers. In addition, uncertainty avoidance modulated activity in medial frontal, precuneus, and left supramarginal areas distinguishing approach decisions towards teachers relative to classmates. Cultural intelligence modulated neural responses to classmate approach/avoidance decisions in anterior cingulate and left parietal areas. Overall, functional activities in distinct brain networks reflected different personal socio‐cultural preferences *despite* observed social decisions to interact with others of differential power status. Such findings highlight that social approach or avoidance behaviors towards powerful persons involves differential subjective neural processes possibly involved in computing implicit social utility.

## Graphical abstract

Social interaction is highly individualized to socio‐cultural preferences and power status. While individual socio‐cultural preferences mitigate social interactions involving persons of power, there is limited empirical data on the underlying neural correlates. To address this, we asked participants to decide whether they were willing to engage in social activities involving their teachers (higher power status), classmates (equal power status), or themselves (control) while functional brain images were acquired. Questionnaires assessed participants’ preferences for power distance and uncertainty avoidance, and cultural intelligence. Our findings suggest that social approach or avoidance behaviors towards powerful persons involves differential subjective neural processes possibly involved in computing implicit social utility.

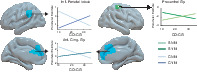

## 1 INTRODUCTION

Social power, the degree to which an agent influences other individuals, is a key component of social decisions to approach or avoid others (Keltner et al., 2003; Magee & Smith, 2013). Because of our limited ability to predict how more powerful others behave or control their larger magnitudes of influence over us, decisions to socialize with them are more uncertain and riskier compared to decisions about peers. Indeed, to arbitrate social contexts involving persons of higher than lower power status, greater neural activity is engaged (Breton et al., 2014; Watanabe & Yamamoto, 2015) across brain regions including the amygdala (Kumaran et al., 2012), medial temporal (Tavares et al., 2015), parietal, lateral and medial frontal, and striatal (Farrow et al., 2011; Marsh et al., 2009; Muscatell et al., 2012; Zink et al., 2008) areas, which implicate a broad range of operations involved in affective reactions, cognitive integration, reward prediction, and motivation. Importantly, there exists individual variability in decisions about whether or not to interact with more powerful persons. More egalitarian persons might forgo interacting with more powerful persons because the potential need to acquiesce personal choice is not preferred. Yet, one might view that being in favor with powerful persons might yield greater benefits. Such individual differences indicate that the power status of the other is not the only determinant of social choices and that personal socio‐cultural preferences also mitigate subjective perceptions of outcome utility and the neural processes involved in social decisions (Adolphs, 2003; Singer, 2012).

In this study, we sought to characterize the neural correlates underlying three socio‐cultural preference dimensions identified in behavioral studies that simultaneously but distinctively influence social perceptions and interaction behaviors with persons of differential power status: power distance (PD; the acceptance level for unequal power distributions), uncertainty avoidance (UA; discomfort with social risk), and cultural intelligence (CQ; adaptability to diverse sociocultural contexts) (Hofstede, 2001; Sharma, 2010). Studies have documented greater demand for fair interactions with more powerful people in those preferring the equalization of social power (indexed by low PD scores) but a focus on the social utility of interactions with powerful people in those who prefer hierarchical social structure (indexed by high PD scores) (Farh et al., 2007; Kirkman et al., 2009; Tyler et al., 2000; Vidyarthi et al., 2014). In addition, approach behaviors are reduced and avoidance increased to the extent a person harbors high UA and feels uncomfortable in unstructured or unpredictable social contexts (Gudykunst & Ting‐Toomey, 1988; Hofstede, 2011; Merkin, 2006). Finally, social approach and collaborative behaviors are also enhanced by psychological flexibility in perceiving and strategizing across various social contexts reflected in higher CQ (Ang et al., 2007, 2019; Gozzoli & Gazzaroli, 2018; Heyes et al., 2020), which also consists of four sub‐dimensions including the cognitive (knowledge of others' cultures), metacognitive (self‐perspective on cultural knowledge), motivational (impetus to engage with different cultures), and behavioral (ability to adjust communicative styles) components (Gozzoli & Gazzaroli, 2018; Heyes et al., 2020). Such distinct social interaction behaviors associated with socio‐cultural preferences motivated us to better understand how these endogenous individual differences modulate neural processing of decisions to approach and avoid interactions with others of differential power status.

In addition to the brain areas above that are generally sensitive to social power differences, right frontal functional responses when passively viewing faces of more powerful persons are modulated by beliefs about social hierarchy (Ligneul et al., 2017). Taking these together, we considered that individuals who evince higher PD, lower UA, or higher CQ generally harbor greater sensitivity to the utility of interacting with persons of higher power. When choosing to approach relative to avoiding more powerful persons, those with such socio‐cultural preferences should show higher activity in brain areas implicated in social value processing. By contrast, lower PD, higher UA, or lower CQ correspond with reduced social incentives regarding interactions with more powerful persons. Those with such socio‐cultural preferences should evince lower neural social value processing activity associated with choosing to approach relative to avoiding more powerful persons. Nevertheless, there remains sparse empirical evidence on how socio‐cultural preferences modulate neural processing during social approach and avoidance decisions.

**FIGURE 1:**
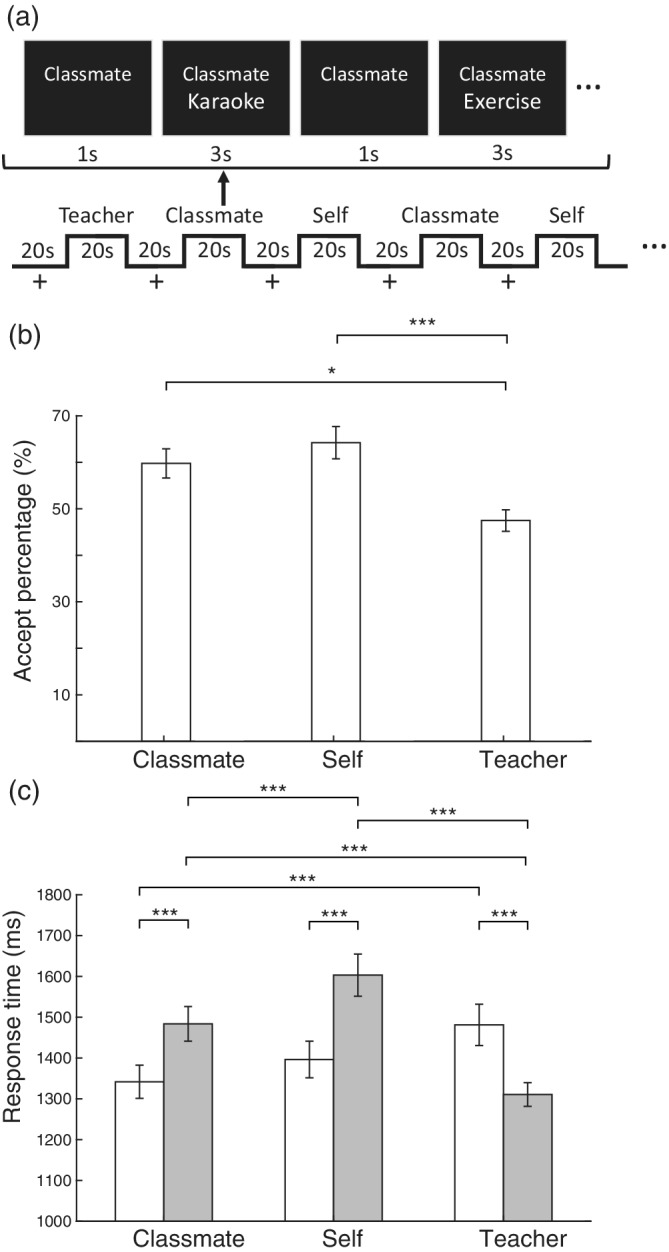
(a) Schematic of the social decision task (SDT) blocked‐design fMRI paradigm. In each trial (4 s), text indicating one of the three target persons (teacher, classmate, or self) was presented on the screen and remained, as text indicating different activities (e.g., karaoke, exercise, etc.) appeared below subsequently. There were five trials depicting different activities in each of the eight blocks per target person condition. On presentation of each activity, participants indicated within 3 s whether they would accept or reject engaging in the stated activity with the target persons using assigned button presses. Target person text for the teacher and classmate conditions were actual names of a professor and a classmate provided by each participant prior to the MRI experiment, respectively. (b) Bar graph showing mean percentages of acceptances in behavioral responses during the SDT. (c) Bar graph showing mean response times of acceptances (white) and rejections (gray) in the Social Decision Task. Error bars signify standard errors. *FDR‐*p* < .05; **FDR‐*p* < .01; ***FDR‐*p* < .005 (FDR, false discovery rate)

To fill this literature gap, we applied a Social Decision Task (SDT) functional magnetic resonance imaging (fMRI) experiment (Figure 1a) in which university students chose to accept or reject engaging in different common campus activities (e.g., karaoke, exercise, choosing research topics, etc.; Table S1) with a specific target person. The target person was either a teacher (of higher social power status), a classmate (an acquaintance in class having similar social status), or the self (control reference). Target persons in the Teacher and Classmate conditions were actual persons participants named prior to starting the experiment. Thus, participants were instructed to consider engaging in the listed activity with the real‐life persons they named accordingly. In addition, participants indicated their familiarity and power distances with respect to the target persons and completed questionnaires assessing their socio‐cultural preferences in PD, UA, and CQ. We expected that students should generally be more willing to participate in social activities with classmates than teachers. Correspondingly, decisions to interact with teachers should require greater neural integration than decisions to interact with classmates. Critically, we were interested in how the three different dimensions of personal preferences would further modulate brain activity during a given behavioral decision regarding socializing with teachers or classmates. In general, we expected socio‐cultural preferences to modulate neural activity more when deciding to accept or reject interacting with teachers but less so for social decisions regarding classmates.

## 2 MATERIALS AND METHODS

### 2.1 Participants

Twenty‐six healthy Taiwanese undergraduate and graduate students (11 females) between 20 and 35 years old (mean age = 23.21 years; SD = 3.32) participated in this study. All participants were right‐handed with normal or corrected to normal vision. None of the participants had a history of neurological or psychological disorders. Study procedures and tasks were approved by the Internal Review Board at Taiwan National Health Research Institute and National Taiwan Normal University. All participants provided written informed consent before testing and were remunerated for their participation in this study.

### 2.2 Assessment of personal socio‐cultural preferences

Before scanning, participants completed 7‐point Likert scale questionnaires assessing their socio‐cultural preferences. The PD scale contains 5 items assessing acceptance of unequally distributed power (Sharma, 2010). The UA scale consists of five items assessing a person's intolerance for uncertainty and ambiguity in unstructured situations (Shkurko, 2020). The CQ scale includes 20 items assessing a person's cultural adaptability, with four sub‐dimensions: metacognition (CQ‐MC), behavior (CQ‐BH), cognition (CQ‐CG), and motivation (CQ‐MT) (Kirkman et al., 2009).

### 2.3 Social decision task

Before the SDT, participants named a teacher (i.e., an authoritarian figure) and a peer (i.e., a classmate they were acquainted with) whom they interacted with on campus. Then, participants further indicated the subjectively perceived social distances and relative power differences between themselves and the two target persons. Higher ratings indicate lower familiarity and higher power differences, respectively. In the SDT, text stimuli for the three conditions (i.e., Teacher, Classmate, and Self) were presented to participants in the scanner (Figure 1, panel a). Each block‐designed conditions contained 43 common on‐campus social activities (Table S1). The task was presented using E‐Prime 2.0 software (Psychology Software Tools, Inc., Sharpsburg, PA, USA) and projected onto a screen at the back of the scanner, which participants viewed using a mirror mounted on the head coil.

Each of the two fMRI runs lasted 484 s long and contained four blocks per condition, with five trials per block. A 20‐s fixation stimulus (white cross on a black background) separated each block. In each run, a 14‐s fixation preceded the beginning of the first block, and a 10‐s fixation terminated the last block. Block condition order was pseudo‐randomized so that no condition was repeated more than three times consecutively. Participants underwent a brief practice session prior to entering the scanning room consisting of nine target person‐social activity pairs (three pairs per target) not used in the main experiment.

### 2.4 Brain imaging parameters

MR experiments were performed on a 3 T MRI system (Prisma, Siemens Healthcare, Erlangen, Germany) using a 20‐channel head radio‐frequency receive coil. A rapid T1 magnetization prepared rapid acquisition gradient echo sequence with the following parameters, repetition time (TR) of 2000 ms, echo time (TE) of 2.3 ms, flip angle of 8°, field‐of‐view of 240 mm × 240 mm, matrix size of 256 × 256 and 192 slices, was utilized to acquire high resolution structural images. Each run of blood oxygenation level dependent (BOLD) fMRI scans were acquired using a gradient echo planar imaging sequence with the following parameters: TR of 3000 ms, TE of 32 ms, flip angle of 90°, FOV of 240 mm × 240 mm, matrix size of 96 × 96, slice thickness of 2.7 mm, 49 slices and 160 repetitions.

### 2.5 fMRI preprocessing and analysis

fMRI analysis was performed using Statistical Parametric Mapping (SPM12; Wellcome Trust Centre for Neuroimaging, London, UK). For each participant, fMRI data were corrected for head motion and slice‐timing acquisition differences, coregistered to the T1 images, spatially normalized to Montreal Neurological Institute (MNI) template space and spatially smoothed with an 8 mm × 8 mm × 8 mm full‐width at half‐maximum Gaussian kernel. Low‐frequency drifts of BOLD fMRI signals were filtered out by a high‐pass filter with a cutoff‐frequency at 128 s. To account for serial correlations in fMRI time series due to aliased biorhythms, a first‐order autoregressive model was applied in the parameter estimation.

First‐level general linear models were used to estimate whole‐brain voxel‐wise BOLD responses associated with the different target persons (T: Teacher; C: Classmate; S: Self) and response types (Y: Yes; N: No), resulting in six conditions in total (TY, TN, CY, CN, SY, SN). Thus, for each run, six condition onset delta (zero duration) regressors were convolved with the hemodynamic response function and movement regressors were included as covariates. We then generated whole‐brain between‐condition contrasts of neural response estimates for each participant including TY‐TN, TY‐CY, TY‐SY, TN‐CN, TN‐SN, CY‐CN, CY‐SY, CN‐SN, and SY‐SN, and submitted these to group analyses.

Group‐level analyses were conducted based on whole‐brain voxel‐wise regressions. The voxel‐wise regression models included each of the above between‐condition contrasts of neural response estimates as the dependent variable and each of the above seven socio‐cultural preference ratings as the independent variable across separate models. Questionnaire ratings were demeaned before being entered into regression models. The resulting voxel‐wise regression coefficients for the effects of preference ratings indexed the association between the socio‐cultural preference and neural response differences in approaching or avoiding the target person, i.e., teachers or classmates. Voxel coefficients were defined as significant based on a primary voxel *p*‐value of <.001 and a whole‐brain cluster‐level family‐wise error rate of *p*(FWE) < .05 based on 3DClustSim (Han et al., 2019).

## 3 RESULTS

### 3.1 Greater willingness to interact with classmates than teachers in the SDT

For social perceptions regarding named target persons, participants indicated significantly greater familiarity with classmates (*t*(24) = 5.56, FDR‐*p* < .001) and smaller power distances between themselves and classmates (*t*(24) = 13.42, FDR‐*p* < .001) relative to teachers (Table S2). In the SDT, acceptance rates of activities with classmates were higher than acceptance rates for teachers (*t*(24) = 3.26, FDR‐*p* = .012) (Figure 1b). Also, participant decision response times of accepting activities were faster for classmates than teachers (*t*(24) = 4.00, FDR‐*p* = .001). By contrast, rejection decisions were faster for teachers than classmates (*t*(24) = 5.07, FDR‐*p* < .001) (Figure 1c).

### 3.2 Associations between social perceptions of target persons, SDT performances, and socio‐cultural preferences

Socio‐cultural preference ratings are summarized in Table S2. Subjective indications of perceived power status difference between participants and chosen target classmates negatively correlated with CQ (*r* = −0.567, FDR‐*p* = .034). We also note the following associations, albeit they did not survive correction for multiple comparisons. Perceived power status difference with classmates negatively correlated with classmate familiarity (*r* = −0.427, *p* = .033), CQ‐MC (*r* = −0.414, *p* = .040), CQ‐CG (*r* = −0.402, *p* = .046) and CQ‐MO (*r* = −0.534, *p* = .006). Greater familiarity with teachers correlated with higher SDT acceptances of interactions with teachers (*r* = 0.493, *p* = .012). Higher UA correlated with more SDT rejections of classmate interactions (*r* = 0.490, *p* = .013). Finally, higher PD correlated with higher CQ‐CG (*r* = 0.416, *p* = .039).

### 3.3 Greater neural activity when accepting than rejecting interactions with teachers

**FIGURE 2:**
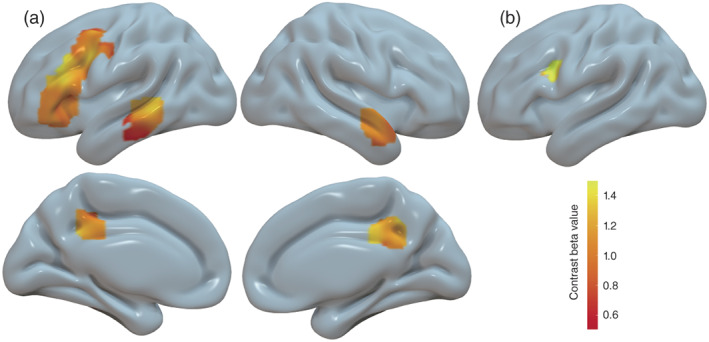
Participants engaged greater neural activity when accepting than when rejecting interactions with teachers. (a) Left frontal lobe, bilateral temporal and precuneus areas showing significant contrast for TY > TN. (b) The left inferior frontal area showing significant contrast for (TY > TN) > (CN > CY). Significance threshold for statistical brain contrast overlays was set at a primary voxel level *p* < .001, and whole‐brain cluster‐wise family wise error (FWE) rate of *p* < .05

Supporting the SDT behavioral responses and past literature, when accepting relative to rejecting social interactions with teachers, participants engaged higher neural activity across the precuneus, bilateral temporal, and left middle and inferior frontal areas (Figure 2a and Table S3). No significant differences of neural activity were observed in the cerebrum between accepting and rejecting social engagements with Classmates (CY > CN) or by Self (SY > SN). Further, direct comparison of the neural engagement differences between accepting and rejecting social engagements with teachers relative to that with classmates revealed significantly greater contrast in the left inferior frontal gyrus (Figure 2b). These findings are generally consistent with the notion that the utility of approaching persons of higher power involves assessments of greater risk compared to that of avoiding them.

### 3.4 Power distance preference modulates right hemisphere response differences to accepting versus rejecting interactions with teachers

**FIGURE 3:**
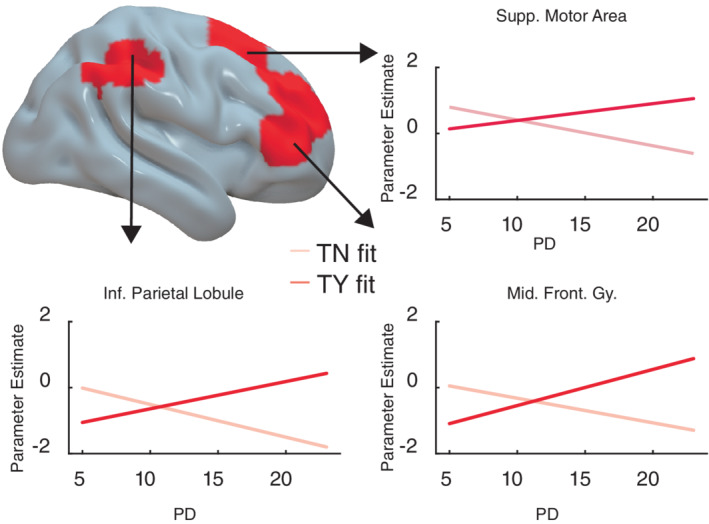
Statistical map overlay showing brain areas in which power distance (PD) scores were significantly associated with the TY—TN (accepting versus rejecting interacting with teachers) neural response contrast estimates. Statistical threshold was set at a primary voxel level *p* < .001, and whole‐brain cluster‐wise family wise error (FWE) rate of *p* < .05. Brain areas observed were all in the right hemisphere, including the supplementary motor, inferior parietal, and middle frontal areas. Linear fit lines show the different direction of neural responses in TN and TY conditions respectively as PD increases. Lighter red lines show decreasing trend of neural responses in TN condition as PD increases, whereas darker red lines show increasing trend of neural responses in TY condition as PD increases within regions of interest. See Figure S1 for individual data points for TY and TN responses

Crucially, socio‐cultural preferences further modulated neural response differences in making approach relative to avoidance decisions regarding teachers. Specifically, higher PD scores were associated with greater neural response differences between accepting and rejecting interactions with teachers (TY > TN) in the right supplementary motor, middle frontal and inferior parietal areas (Figure 3, Table 1, and Figure S1). By contrast, social decision neural response contrasts for Classmates (CY > CN) and non‐social Self (SY > SN) conditions did not evince any significant associations with PD at the voxel‐wise level. To visualize these neurobehavioral associations, we defined regions of interest (ROIs) from the above brain areas and extracted individual neural responses when accepting (TY) and rejecting (TN) social engagements with teachers. As can be seen in Figure 3 (see also Figure S1), neural activities in these right hemisphere ROIs were higher for TN than TY in those with lower PD but were higher for TY than TN in those with higher PD. These right hemisphere activity variations reflect internal strategic differences in the neural processing of a given social decision as a function of PD preferences beyond general left hemisphere processing of approach or avoidance behavior towards others of higher social power. No other preference measures significantly modulated neural TY versus TN contrast responses.

**TABLE 1:** List of MNI (Montreal neurological institute; *x*, *y*, *z*) coordinates and Brodmann's areas (BA) of peak voxels showing significant associations between neural responses during the social decision task and power distance (PD), uncertainty avoidance (UA), cultural intelligence (CQ), and cultural cognition (CQ‐CG) socio‐cultural preferences

Association	Brain region	BA	*x*	*y*	*z*	*K*	*T*
(TY > TN) ~ PD	R Supp. Motor Area	8	16	14	60	441	6.55
	R Mid. Front. Gy.	46	18	50	20	578	5.62
	R Inf. Parietal Lobule	40	56	−40	48	377	4.66
(TY > SY) ~ PD	R Mid. Front. Gy.	45	38	40	6	352	5.22
	R Ins.	48	48	0	−2	1013	7.21
	L Ins.	48	−50	12	−8	284	5.06
(CY > SY) ~ PD	L Sup. Temp. Gy.	22	−62	−4	10	466	5.58
	L Supramarginal Gy.	48	−56	−38	26	144	5.22
	R Inf. Front. Gy.	47	42	38	−4	151	5.01
	R Rolandic Oper.	48	48	−22	20	219	4.83
	L Mid. Cing. Gy.	24	−8	8	34	149	4.57
(TY > CY) ~ UA	Ant. Cing. Gy.	24	−2	36	12	603	−5.72
	Precuneus	7	0	−72	38	299	−5.42
	L Med. Sup. Front. Gy.	10	−2	60	16	290	−5.14
	R Sup. Front. Gy.	8	24	32	56	349	−5.13
	L Supramarginal Gy.	40	−56	−50	34	174	−5.01
	Mid. Cing. Gy.	23	6	−32	26	219	−4.46
(TY > CY) ~ CQ	R Mid. Occ. Gy.	18	32	−84	12	604	−5.76
	L Mid. Occ. Gy.	18	−30	−90	0	231	−4.59
(CY > CN) ~ CQ‐CG	L Inf. Parietal lobule	40	−52	−38	38	144	5.98
	Ant. Cing. Gy.	24	6	14	24	472	5.83
(SY > SN) ~ CQ‐CG	R Precentral Gy.	4	38	−26	62	82	−5.01

*Note*: Neural responses were the contrasts of functional activity during accepting (TY) or rejecting (TN) interacting with teachers, agreeing (CY) or rejecting (CN) interacting with classmates, and accepting (SY) or rejecting (SN) interacting by self. Significance criteria were set as a primary voxel threshold *p* < .001 and whole‐brain cluster‐wise family‐wise error (FWE) rate of *p* < .05. No other associations yielded significant brain areas.

Interestingly, higher PD scores were also associated with more positive neural response contrast between accepting interactions with teachers versus self (TY > SY) in bilateral insula cortices, and right middle frontal gyrus and classmates versus self (CY > SY) in left temporal, and supramarginal, right frontal, and insula, and middle cingulate areas (Table 1). These results consistently reflect higher neural engagement especially in right frontal/insula regions for accepted activities with others relative to the Self particularly for those with higher PD (no reverse contrast effects were observed). In addition, we also performed a whole‐brain analysis that considered PD with the other socio‐cultural preference ratings simultaneously in an omnibus model for the TY > TN contrast (Figure S2). In this omnibus model, the right middle frontal and inferior parietal ROIs were absent while the right supplementary motor area remained. Finally, we also examined a whole‐brain model considering individual mean RTs of responses as a covariate for the TY > TN contrast (Figure S3). Similar to the omnibus model, the right middle frontal and inferior parietal ROIs were absent while the right supplementary motor area remained.

### 3.5 Uncertainty avoidance and cultural intelligence modulate neural response differences in accepting interactions with teachers versus classmates

**FIGURE 4:**
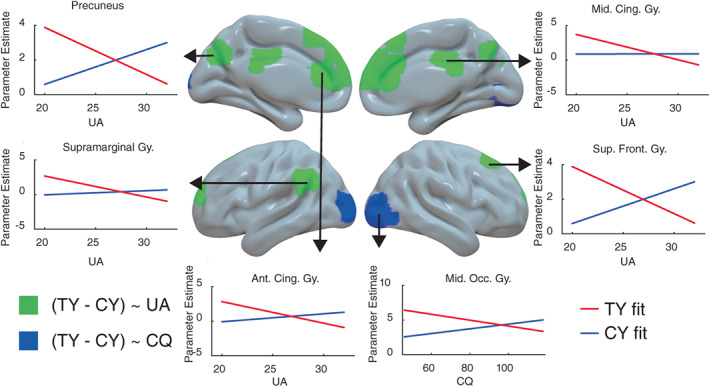
Statistical map overlays showing brain areas in which uncertainty avoidance (UA, green) and cultural intelligence (CQ, blue) scores were significantly associated with the TY‐CY (accepting interacting with teachers versus classmates) neural response contrast estimates. Lateral and medial views of significant areas for both hemispheres are shown. Statistical threshold was set at a primary voxel level *p* < .001, and whole‐brain cluster‐wise family wise error (FWE) rate of *p* < .05. UA modulated brain responses in precuneus, middle cingulate gyrus, left supramarginal gyrus, superior frontal gyrus, anterior cingulate gyrus, whereas CQ modulated brain responses in bilateral occipital areas. Linear fit lines indicate opposite trends between TY (red fit line) and CY (blue fit line) as UA or CQ increases. See Figure S4 for individual data points for accept and reject responses

Higher UA scores were associated with more negative neural response contrast between accepting interactions with teachers versus classmates (TY > CY) in the anterior medial frontal, anterior and middle cingulate, precuneus, and left supramarginal areas (Figure 4, Table 1, and Figure S4). In these ROIs, neural activity for accepting socializing with teachers (TY) relative to classmates (CY) increased as UA decreased. Thus, low UA participants enhanced neural processes in these brain areas, more than those with high UA, when approaching persons of higher power compared to peers. Higher CQ scores were associated with more negative neural response contrasts of TY versus CY in bilateral middle occipital gyri (Figure 4 and Table 1). Specifically, the difference in neural activity between CY and TY increased as CQ increased. Overall, UA and CQ scores modulated neural response to TY decisions that involved distinct brain areas from those involved in the influence of PD. PD did not significantly modulate TY versus CY neural response contrasts. Both omnibus social preference (Figure S2) and RT‐covariate (Figure S3) whole‐brain models of the TY > CY contrast showed minimal differences relative to the above results.

### 3.6 Cultural cognition modulates neural response differences between accepting versus rejecting interactions with classmates and self

**FIGURE 5:**
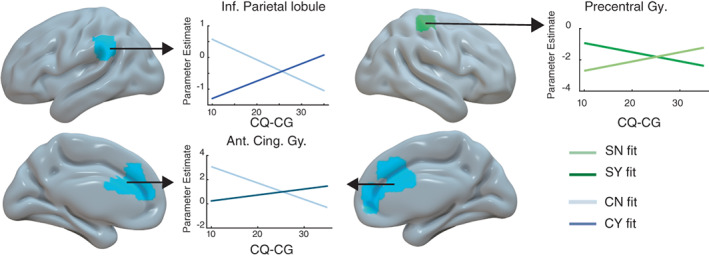
Statistical map overlay showing brain areas in which the cognitive dimension of cultural intelligence scores were significantly associated with the CN‐CY (rejecting versus accepting interacting with classmates, cyan) and SY‐SN (accepting versus rejecting attending activities alone, green) neural response contrast estimates. Statistical threshold was set at a primary voxel level *p* < .001, and whole‐brain cluster‐wise family wise error (FWE) rate of *p* < .05. CQ‐CG modulated CN‐CY brain response contrasts in left inferior parietal lobule and anterior cingulate gyrus and SY‐SN brain response contrasts in the right precentral gyrus. Linear fit lines show the different directions of neural responses as CQ‐CG increases. Lighter blue lines show decreasing trend of neural responses in CN condition as CQ‐CG increases, whereas darker blue lines show increasing trend of neural responses in CY condition as CQ‐CG increases within the ROIs. The lighter green line shows increasing trend of neural responses in SN condition as CQ‐CG increases, whereas the darker green line shows decreasing trend of neural responses in SY condition as CQ‐CG increases within the ROIs. See figure S5 for individual data points for accept and reject responses for the classmate condition

Although participants' neural responses for acceptance and rejection decisions regarding classmates (CY > CN) and self (SY > SN) did not significantly differ on average, socio‐cultural preferences still modulated neural processing of these decisions. Specifically, higher CQ‐CG was associated with greater neural response for CY than CN decisions in anterior cingulate and left inferior parietal areas (Figure 5, Table 1, and Figure S5). We note that these brain areas were adjacent but distinct from those identified in association with UA above. In these ROIs, neural activities were lower for CY relative to CN decisions in those with lower CQ‐CG but higher for CY than CN in high CQ‐CG participants. Also, higher CQ‐CG was associated with greater neural response for SY than SN decisions in right precentral gyrus (Figure 5 and Table 1). Here, neural activities were lower for SN than SY decisions in those with lower CQ‐CG but higher for SN than SY in high CQ‐CG participants. No other associations with neural responses were found for the other aspects of CQ.

### 3.7 Different voxel‐overlap patterns in brain areas showing modulatory effects of PD, UA, and CQ

Finally, we quantified the degree of overlap and distinctiveness of the brain areas showing differential social preference modulation of neural processing of social interaction decisions identified above. Voxel overlap for all the above whole‐brain effects using the same whole‐brain threshold criterion are illustrated in Figure S6 and listed in Table 2. Three areas showed overlap for the effects of UA with CQ‐CG (anterior cingulate gyrus and left inferior parietal lobule) and with PD (right superior frontal gyrus). Interestingly, the right middle frontal gyrus showed overlapping voxel engagement for the effect of PD in modulating Teacher‐related decisions (TY > TN), acceptance of activities with Teachers versus by the Self (TY > SY), and with Classmates versus by the Self (CY > SY). In addition, we interrogated the number of unique voxels in the ROIs listed in Table 1 as voxels that were members of only one association (Table S4). This revealed that the total number of unique voxels (7078) was 92.6% of the total number of voxels across all associations (7641). Overall, the brain regions associated with the modulatory influences of PD, UA, and CQ‐CG on social decision processes are broadly distinct with some minimal overlap.

**TABLE 2:** Brain areas and number of voxels that overlapped between the differential social preference effects (PD, UA, CQ, CQ‐CG) on neural response contrasts for social interaction decisions regarding teachers, classmates, and self (see also Figure S6)

Overlapping associations	Brain region	No. of voxels
(CY > CN) ~ CQ‐CG ∩ (TY > CY) ~ UA	Ant. Cing. Gy.	37
	L Inf. Parietal lobule	3
(TY > CY) ~ UA ∩ (TY > TN) ~ PD	R Sup. Front. Gy.	14
(TY > SY) ~ PD ∩ (TY > TN) ~ PD	R Mid. Front. Gy.	138
(CY > SY) ~ PD ∩ (TY > TN) ~ PD	R Mid. Front. Gy.	43
(CY > SY) ~ PD ∩ (TY > SY) ~ PD	R Mid. Front. Gy.	111

## 4 DISCUSSION

Social engagement decisions are complex problems that involve integrating across various aspects of psychological information (Adolphs, 2003; Singer, 2012). These social computations and choices lead to various real‐life outcomes including obtaining benefits, missing out on opportunities, circumventing potential hurt, or even suffering harm at the personal and group levels. There is thus an impetus to understand how the human brain processes interpersonal interactions to enhance social experiences and minimize those leading to negative outcomes (Ashkanasy et al., 2014; Hannah et al., 2013). In this study, we demonstrate that there are distinct strategic neural activities related to a person's socio‐cultural perceptions and preferences *despite* a given approach/avoidance decision about persons of higher social power.

Our finding that decisions to interact with teachers are associated with more distinctive left lateral frontal engagement compared with decisions to interact with classmates agrees with neural responses linked to social hierarchical information processing (Farrow et al., 2011; Marsh et al., 2009; Zink et al., 2008). Higher lateral frontal neural activity is generally viewed as reflecting greater regulatory control and attentional selection in information processing required for task performance (Desimone & Duncan, 1995; Miller & Cohen, 2001). Thus, we suggest that increased lateral frontal neural activity when deciding to interact with others who are more powerful may indicate additional information processing that is necessary due to greater social uncertainty (Keltner et al., 2003; Magee & Smith, 2013). Indeed, whereas participants evinced a clear willingness to engage in social interactions with classmates, they were less definitive for social decisions for teachers. In this present study, we further show that left lateral frontal neural activity is higher for approach relative to avoidance decisions with regard to the same powerful persons. For this reason, we consider that left frontal neural activity might be instrumental for the implementation of specific social approach actions towards persons of higher power.

We also found that higher PD preference was associated with higher right frontal neural activity during decisions to accept relative to rejecting interactions with teachers. This finding is consistent with previous studies of social hierarchy (Ligneul et al., 2017), fairness and social norms (Spitzer et al., 2007). Critically, in the ultimatum game, disruption of right dorsolateral prefrontal function induced more selfish decisions (Knoch et al., 2006) and right dorsolateral prefrontal stimulation enhanced normative social behaviors by increasing fairness in economic exchanges (Ruff et al., 2013). Finally, in our previous study, individuals with greater value for hedonism accepted more losing stakes in risky lottery decisions and showed higher right dorsolateral frontal activity as well (Chuang et al., 2020). Integrating these findings, we speculate that higher right and left frontal neural activity when approaching powerful persons reflects greater internal psychological regulation of the social risks and external contextual norms in those who prefer hierarchical social structure. By contrast, higher right but reduced left frontal neural activity when avoiding powerful persons might reflect prioritizing of self‐related motivations (or the de‐prioritizing of higher‐other‐related motivations) in those who are more egalitarian.

PD and UA modulated neural responses during social interaction decisions for teachers across the medial frontal, precuneus, and bilateral inferior parietal areas that are part of the default‐mode network (DMN). Specifically, participants with higher PD or low UA engaged higher neural responses in DMN when accepting compared to rejecting interactions with teachers or accepting interactions with classmates. These regions are associated with coding higher social value (Amodio & Frith, 2006; Bault et al., 2011; Chen et al., 2010; Courtney & Meyer, 2020; Iacoboni et al., 2004; Kim, 2020; Marsh et al., 2009; Radke et al., 2016; Tricomi et al., 2010; Wagner et al., 2016) and self‐related processing (Buckner et al., 2008; Gusnard et al., 2001; Muscatell et al., 2012; Raichle et al., 2001; Spreng et al., 2009). Studies have also reported the sensitivity of the ventromedial area of the DMN in processing uncertainty during decision‐making (Krug et al., 2014; Trudel et al., 2021). Indeed, uncertainty or risk is thought to be an aversive mental state in the brain (Peters et al., 2017) and social uncertainty particularly so (FeldmanHall & Shenhav, 2019; Merkin, 2006). As mentioned, the key consideration with respect to persons of higher power is the social uncertainty or risk surrounding how they might behave (Keltner et al., 2003; Magee & Smith, 2013). Interestingly, evidence suggests that persons who have stronger self‐identity tend to exhibit greater social openness and reduced social anxiety (Kaplan et al., 2015; Whitbourne, 1986). Taking the above together, we speculate that the higher engagement of the DMN in persons with high PD and low UA might reflect interpersonal interaction strategies in the brain under social uncertainty when deciding to interact with persons of higher power. Future studies manipulating the degree of uncertainty associated with interacting with social superiors are needed to shed greater light on specific DMN mechanisms in social decisions involving power differentials.

Our analysis did not find any brain areas with significantly distinctive neural responses between accepting and rejecting classmate interactions. Nevertheless, similar to PD and UA, approach‐related neural responses for classmates were higher than avoidance‐related responses in participants with higher CQ‐CG (the cognitive aspect of cultural intelligence) in medial frontal and left inferior parietal areas. These medial frontal and inferior parietal areas were adjacent to but distinct from those associated with UA. Moreover, the neural responses regarding interactions with classmates were not associated with PD. We reasoned that while the above neural responses do indicate individual differences in the neuropsychological processing of the social value of approaching/avoiding peers, these likely also involve different psychological representations other than social uncertainty to do with the hierarchy of power structures. It is possible that participants with high cultural adaptability view interaction with peers as more self‐relevant or motivating compared to those with low adaptability. We note that these social approach/avoidance neurobehavioral associations were linked to CQ‐CG rather than the generic cultural intelligence or its other sub‐dimensions (see Section 2). Therefore, the influence of individual differences in cultural adaptability on social decisions might stem from a more risk‐neutral cognitive framing of the social contexts. Overall, distinct neural mechanisms underlie social approach/avoidance decisions when the target is a person of higher power status compared to peers.

We found higher general cultural intelligence was associated with higher visual cortex responses when accepting interactions with classmates compared to teachers. We did not expect any visual processing differences in our study that used text stimuli. Interestingly, this result might reflect the imagination of interaction activities with the target person. Those with higher CQ might have constructed more vivid imagery of the social activity, particularly with peers compared to more socially distant targets such as teachers. Such a view would be consistent with the higher acceptance behavior for social interactions with classmates than teachers. We also note the lack of associations between socio‐cultural preference ratings and behavioral responses to teachers in our experimental Social Decision Task. It is possible that the university students in this study exhibited general social conformity in their behavioral decisions to accept or decline interacting with teachers such that individual differences in internal preferences did not explicitly modulate decision behaviors in our simplistic text‐based hypothetical experimental contexts. However, we note that many of our daily social decision processes are in fact based on hypothetical simulation of future consequences as well, of which a select few of those are implemented. Future studies implementing more consequential social decisions regarding more powerful persons might better assess individual differences in decision choice behaviors. Also, studies are needed to examine social preferences effects in social decisions using other samples, for example in work or military contexts, and also considering possible modulation of socioeconomic factors to empirically evaluate generalizability. Nevertheless, we suggest that the relationship between teachers and students investigated in this present study is a valid exemplification of social power effects because the age seniority and pedagogical role of teachers applies greater authority and influence over the students' lives rather than vice versa in the contemporary society. Thus, our results show that for a given social decision to approach or avoid more powerful others, distinct psychological strategies related to individual socio‐cultural preferences are involved, possibly to resolve uncertainty or induce motivation regarding the proposed social interaction behavior. We note that right inferior frontal and inferior parietal areas showing effects of PD when considered in isolation were not present in the omnibus analysis considering PD along with UA and CQ social preference ratings. This suggests some mediating roles of UA or CQ in the effect of PD on TY‐TN, and points to the possible hierarchical nature in the operation of these socio‐cultural constructs. Studies directly manipulating the impact of these separate aspects of social preferences on interaction outcomes are needed to more specifically examine the underlying neuropsychological mechanistic relationships. Indeed, together with the overlapping involvement of right middle frontal gyrus across social decisions involving TY and CY conditions, our findings highlight the right fronto‐parietal area as playing a key role in how individual biases for social power, uncertainty, and adaptability jointly operate when reaching a decision to approach or avoid others of differential power status.

In light of increasingly rapid global social changes, making prompt social decisions in various contexts with people from different social statuses is a critical skill in modern societies. For instance, many organizations rely on complex social structures for their operations (Pilny et al., 2016; Schecter et al., 2018) and management of group dynamics is critical (Berger, 2016; de Oliveira et al., 2015; French Jr. & Raven, 1959). Further, how values about power and uncertainty in social relationships are acculturated such as in educational or familial contexts influences how these constructs operate in future social decisions (Hong & Mallorie, 2004; Patterson, 2014). Our study reveals neural correlates (i.e., right frontal and DMN) underlying decisions to interact with persons of different power status. These distinct neural strategies relate to individual differences in the acceptance of social structure and social motivation, which in turn influences how a person computes social uncertainty and risk when deciding to approach or avoid others. We expect our data characterizing neural mechanisms underlying hidden social computation dynamics to lend key insights towards enhancing human social interaction efficacy.

## AUTHOR CONTRIBUTIONS

Joshua Oon Soo Goh, Li‐Wei Kuo and Wei‐Wen Chang designed research; Jui‐Hong Chien and I‐Tzu Hung performed research; Jui‐Hong Chien, I‐Tzu Hung, and Joshua Oon Soo Goh analyzed data; Joshua Oon Soo Goh, Jui‐Hong Chien and I‐Tzu Hung wrote the article.

## CONFLICT OF INTEREST

The authors declare no competing interest.

## Supporting information


**Data S1** Supporting Information

## Data Availability

The data that support the findings of this study are available from the corresponding author upon reasonable request.
