# Supplementary material for: Personal socio‐cultural preferences modulate neural correlates of decisions to socialize with powerful persons
Source: Hum Brain Mapp. 2022 Jun 4;43(14):4422–32. doi: 10.1002/hbm.25963 (PMC9435004; doi:10.1002/hbm.25963)
Supplement: Supplementary file 1 — Data S1 Supporting Information [file HBM-43-4422-s001.docx]

**Table S1.** Activities used in each condition of the social decision task

| Teacher | Classmate | Self |
| --- | --- | --- |
| Failing a class | Failing a class | Failing a class |
| Grade too low | Grade too low | Grade too low |
| Error in test score | Error in test score | Error in test score |
| Going on a trip | Going on a trip | Going on a trip |
| Relationship break up | Relationship break up | Relationship break up |
| Playing basketball | Playing basketball | Playing basketball |
| Applying for scholarship | Applying for scholarship | Applying for scholarship |
| Considering studying abroad | Considering studying abroad | Considering studying abroad |
| Difficult homework | Difficult homework | Considering internship |
| Transferring course credit | Transferring course credit | Difficult homework |
| Argument with a friend | Argument with a friend | Argument with a friend |
| Visiting an art show | Visiting an art show | Visiting an art show |
| Watching a movie | Watching a movie | Watching a movie |
| Seeing a doctor | Seeing a doctor | Seeing a doctor |
| Barbeque | Barbeque | Barbeque |
| Missed a test | Missed a test | Missed a test |
| Research topic discussion | Research topic discussion | Karaoke |
| Karaoke | Karaoke | Shopping |
| Requesting recommendation letter | Requesting recommendation letter | Lecture was too fast |
| Lecture was too fast | Lecture was too fast | Visiting the night market |
| Visiting the night market | Visiting the night market | Too much homework |
| Too much homework | Too much homework | Considering hiatus from school |
| Considering hiatus from school | Considering hiatus from school | Auditing classes |
| Auditing classes | Auditing classes | Withdrawing from class |
| Withdrawing from class | Withdrawing from class | Choosing research topics |
| Changing majors | Changing majors | Changing majors |
| Preparing for interview | Preparing for interview | Preparing for interview |
| New year's eve celebration | New year's eve celebration | New year's eve celebration |
| Taking classes at another university | Taking classes at another university | Exercise |
| Exercise | Exercise | Writing a paper |
| Group purchase activity | Group purchase activity | Birthday celebration |
| Writing a paper | Writing a paper | Asking sick leaves |
| Birthday celebration | Birthday celebration | Tuition fee difficulty |
| Asking sick leaves | Asking sick leaves | Choosing clubs |
| Tuition fee difficulty | Tuition fee difficulty | Choosing graduate schools |
| Choosing clubs | Choosing clubs | High stress |
| Choosing graduate schools | Choosing graduate schools | Not understanding class materials |
| High stress | High stress | Attending seminars |
| Not understanding class materials | Not understanding class materials |  |

**Table S2.** Mean and standard deviation (SD) values of all ratings and scales.

| Scale | PD | UA | CQ-  MC | CQ-  CG | CQ-  MO | CQ-  BH | TPD | TF | CPD | CF |
| --- | --- | --- | --- | --- | --- | --- | --- | --- | --- | --- |
| Mean | 11.28 | 26.56 | 21.36 | 24.92 | 22.32 | 24.24 | 4.12 | 3.64 | -0.16 | 2.24 |
| SD | 4.57 | 3.45 | 3.20 | 6.45 | 5.99 | 5.75 | 0.86 | 1.13 | 1.35 | 0.91 |

Note: PD, power distance scale; UA, uncertainty avoidance scale; CQ-MC, metacognition dimension in culture intelligence scale; CQ-MC, metacognition dimension in culture intelligence scale; CQ-CG, cognition dimension in culture intelligence scale; CQ-MO, motivation dimension in culture intelligence scale; CQ-BH, behavior dimension in culture intelligence scale; TPD, subjective indication of power status difference between participants and chosen target teachers; TF, subjective indication of familiarity with chosen target teachers; CPD, subjective indication of power status difference between participants and chosen target classmates; CF, subjective indication of familiarity with chosen target classmates;

**Table S3.** Activation table listing peak MNI (Montreal Neurological Institute) coordinates and Brodmann's areas (BA) of voxels showing significant neural responses in contrasts during agreeing (TY) or rejecting (TN) interacting with Teachers and agreeing (CY) or rejecting (CN) interacting with Classmates. Significance criteria were set as a primary voxel threshold p < 0.001 and whole-brain cluster-wise family-wise error (FWE) rate of p < 0.05.

| Contrast | Region | | BA | x | y | z | k | T |
| --- | --- | --- | --- | --- | --- | --- | --- | --- |
| TY > TN | L Precentral | | 6 | -36 | 4 | 48 | 303 | 4.37 |
|  | R Mid. Temp. Gy. | | 21 | 62 | 2 | -10 | 284 | 4.32 |
|  | L Inf. Front. Gy. |  | 45 | -56 | 26 | 18 | 380 | 4.28 |
|  | L Mid. Temp. Gy. | | 20 | -52 | -26 | -16 | 168 | 4.17 |
|  | Mid. Cing. Gy. |  | 23 | -4 | -38 | 38 | 229 | 3.92 |
| (TN > TY) – (CY > CN) | L Inf. Front. Gy. |  | 44 | -40 | 4 | 24 | 103 | 4.18 |

**Table S4.** Number of unique (non-overlapping) voxel in brain regions shown in Table 1 using overlapping voxel numbers in Table 2.

| Association | Brain Region | No. of unique voxels |
| --- | --- | --- |
| (TY > TN) ~ PD | R Supp. Motor Area | 427 |
|  | R Mid. Front. Gy. | 440 |
|  | R Inf. Parietal Lobule | 377 |
| (TY > SY) ~ PD | R Mid. Front. Gy. | 146 |
|  | R Ins. | 1013 |
|  | L Ins. | 284 |
| (CY > SY) ~ PD | L Sup. Temp. Gy. | 466 |
|  | L Supramarginal Gy. | 144 |
|  | R Inf. Front. Gy. | 40 |
|  | R Rolandic Oper. | 219 |
|  | L Mid. Cing. Gy. | 149 |
| (TY > CY) ~ UA | Ant. Cing. Gy. | 566 |
|  | Precuneus | 299 |
|  | L Med. Sup. Front. Gy. | 290 |
|  | R Sup. Front. Gy. | 335 |
|  | L Supramarginal Gy. | 171 |
|  | Mid. Cing. Gy. | 219 |
| (TY > CY) ~ CQ | R Mid. Occ. Gy. | 604 |
|  | L Mid. Occ. Gy. | 231 |
| (CY > CN) ~ CQ-CG | L Inf. Parietal lobule | 141 |
|  | Ant. Cing. Gy. | 435 |
| (SY > SN) ~ CQ-CG | R Precentral Gy. | 82 |


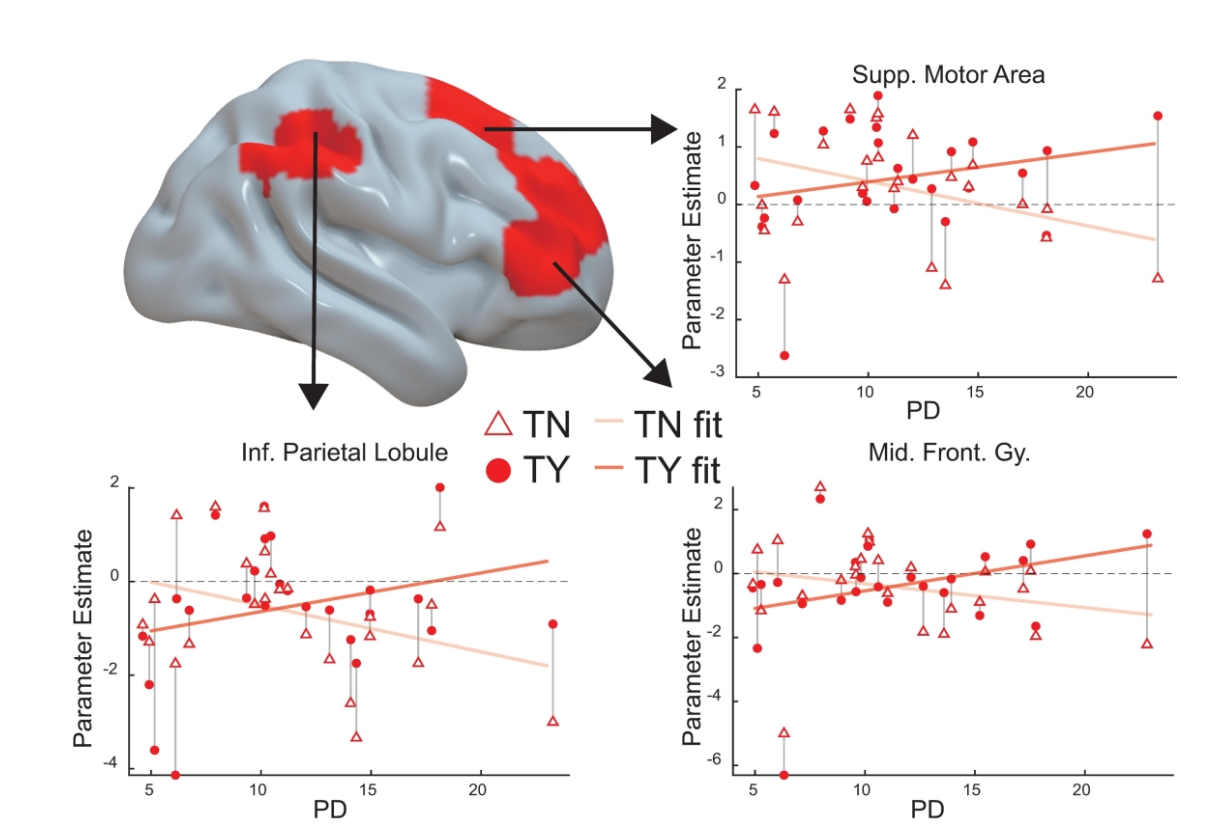


Fig. S1. Individual data for those shown in Fig. 3. Statistical map overlay showing brain areas in which power distance (PD) scores were significantly associated with the TY – TN (accepting vs. rejecting interacting with teachers) neural response contrast estimates. Statistical threshold was set at a primary voxel level p < 0.001, and whole-brain cluster-wise family wise error (FWE) rate of p < 0.05. Brain areas observed were mostly in the right hemisphere, including the supplementary motor, inferior parietal, and middle frontal areas. Unfilled triangles (TN) and filled circles (TY) connected by grey lines indicate neural response estimates of each participant in regions of interest defined from the peak contrast voxels of the above three brain areas. Linear fit lines are added in scatter plots to better show the different direction of neural responses in TN and TY conditions respectively as PD increases. Lighter red lines show decreasing trend of neural responses in TN condition as PD increases, whereas darker red lines show increasing trend of neural responses in TY condition as PD increases within regions of interest.


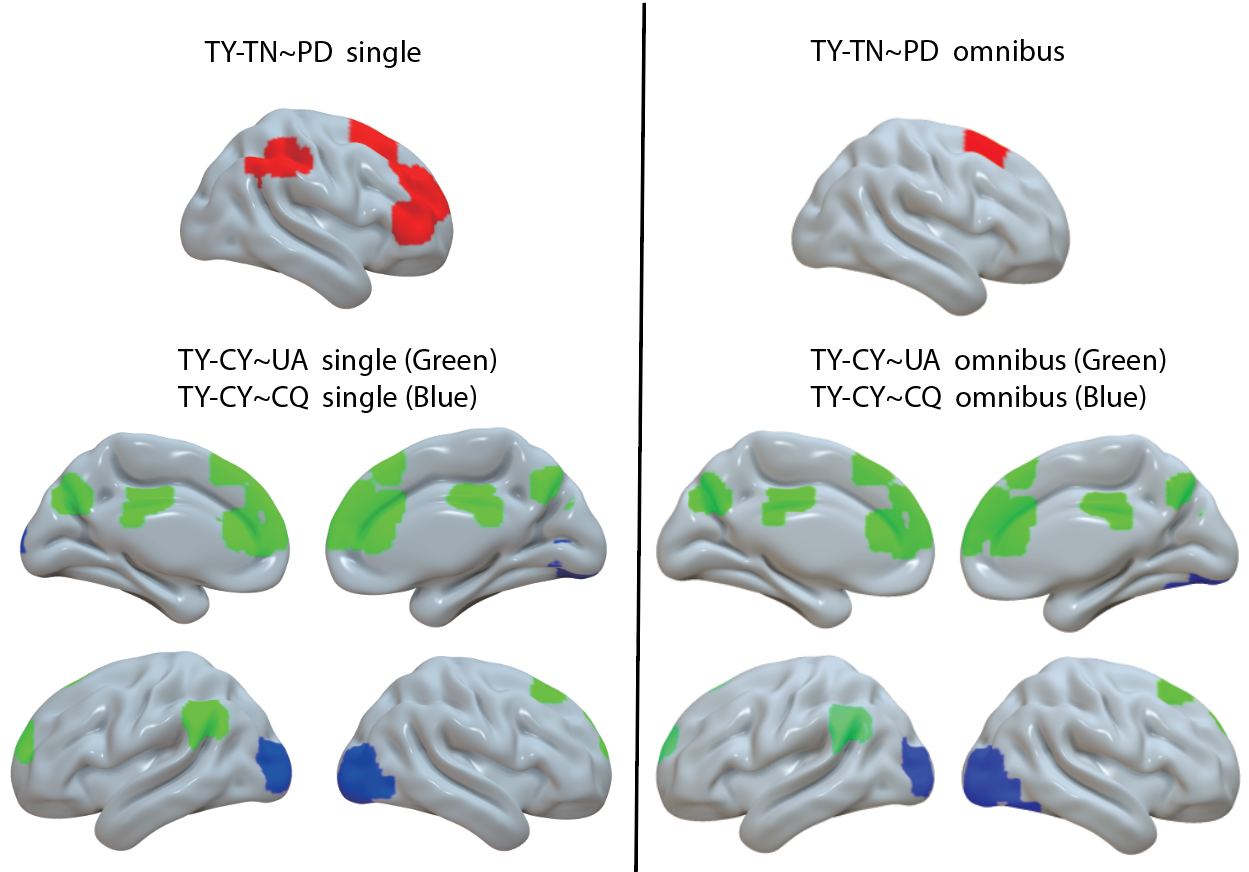


Fig. S2. Comparison of statistical maps in SPM models socio-cultural preferences ratings in three separate models or all three ratings (omnibus) simultaneously in one model. Single rating model results are shown on the left for convenience of comparison. These are the same as statistical maps shown in Fig. S1 and 4. Only the PD effect was somewhat reduced in the omnibus model but still consistent with the original result. The rest of the effects remained mostly similar. Significance thresholds are set at a primary voxel level p < 0.001, and whole-brain cluster-wise family wise error (FWE) rate of p < 0.05.


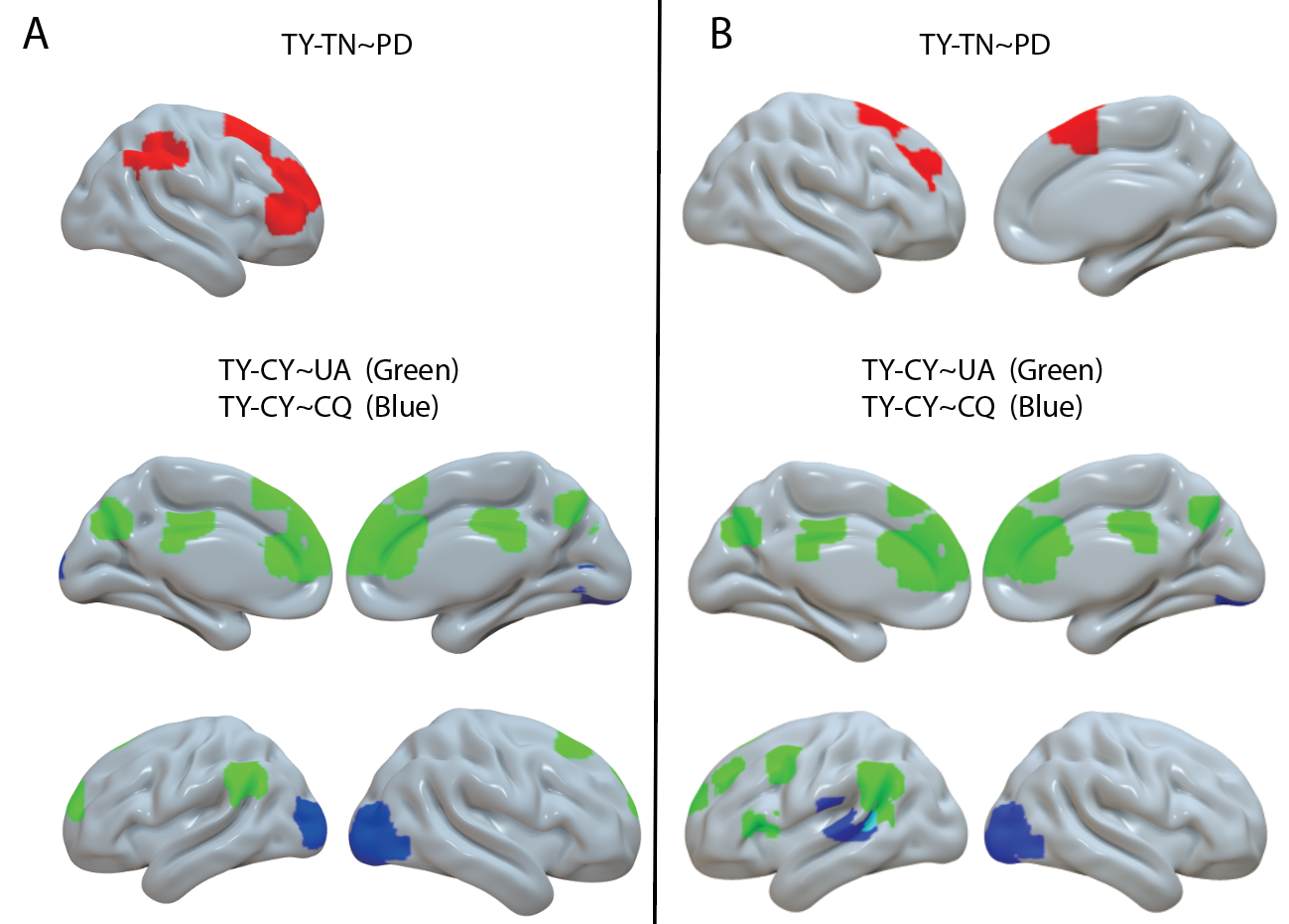


Fig. S3. Comparison of statistical maps with and without including individual mean response times (RT) as covariates in the SPM 2^nd^-level group analysis. (A) Results of statistical maps from models without using RT for convenience of comparison. These are the same as statistical maps shown in Fig. S1 and 4. (B) Results of statistical maps from models including mean RT as covariates. Significance thresholds are set at a primary voxel level p < 0.001, and whole-brain cluster-wise family wise error (FWE) rate of p < 0.05.


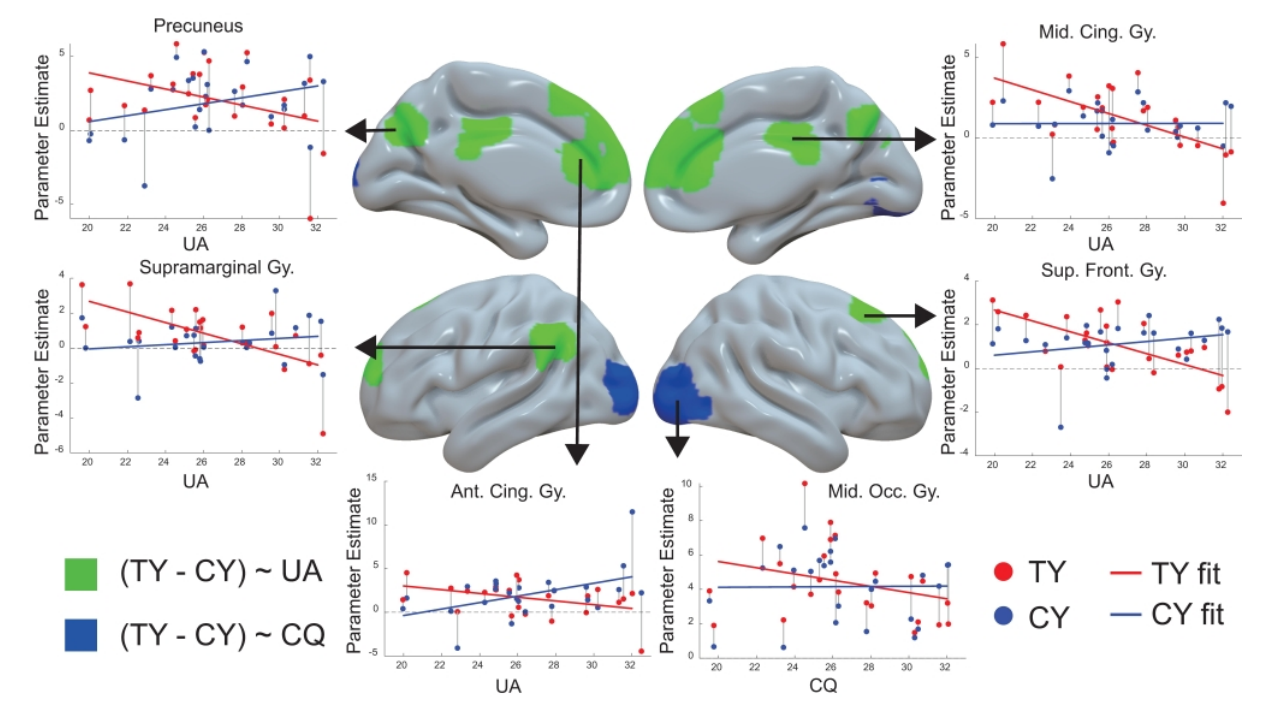


Fig. S4. Individual data for those shown in Fig. 4. Statistical map overlays showing brain areas in which uncertainty avoidance (UA, green) and cultural intelligence (CQ, blue) scores were significantly associated with the TY – CY (accepting interacting with teachers vs. classmates) neural response contrast estimates. Lateral and medial views of significant areas for both hemisphere are shown. Statistical threshold was set at a primary voxel level p < 0.001, and whole-brain cluster-wise family wise error (FWE) rate of p < 0.05. UA modulated brain responses in precuneus, middle cingulate gyrus, left supramarginal gyrus, superior frontal gyrus, anterior cingulate gyrus, whereas CQ modulated brain responses in bilateral occipital areas. Red circles (TY) and blue circles (CY) connected by gray lines indicate neural response estimates of each participant in regions of interest defined from the peak contrast voxels within respective brain areas marked in. Linear fit lines in scatter plots indicate opposite trends between TY (red fit line) and CY (blue fit line) as UA or CQ increases.


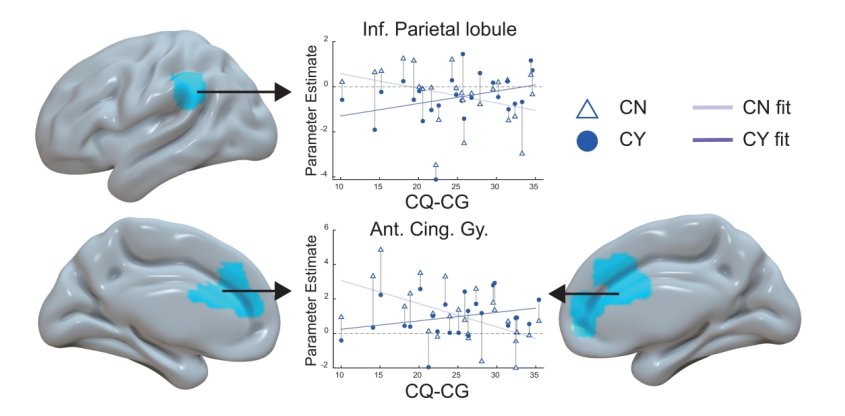


Fig. S5. Individual data for those shown in Fig. 5. Statistical map overlay showing brain areas in which the cognitive dimension of cultural intelligence (CQ-CG, cyan) scores were significantly associated with the CN – CY (rejecting vs. accepting interacting with classmates) neural response contrast estimates. Statistical threshold was set at a primary voxel level p < 0.001, and whole-brain cluster-wise family wise error (FWE) rate of p < 0.05. CQ-CG modulated brain responses in left inferior parietal lobule and anterior cingulate gyrus. Scatter plots show neural response estimates within left inferior parietal lobule and anterior cingulate gyrus vs. CQ-CG. Unfilled triangles (CN) and filled circles (CY) connected with gray lines indicate neural response estimates from each participant in regions of interest defined from the peak contrast voxels of the above two brain areas. Linear fit lines are added in scatter plots to better show the different direction of neural responses in CN and CY conditions respectively as CQ-CG increases. Lighter blue lines show decreasing trend of neural responses in CN condition as CQ-CG increases, whereas darker blue lines show increasing trend of neural responses in CY condition as CQ-CG increases within regions of interest.


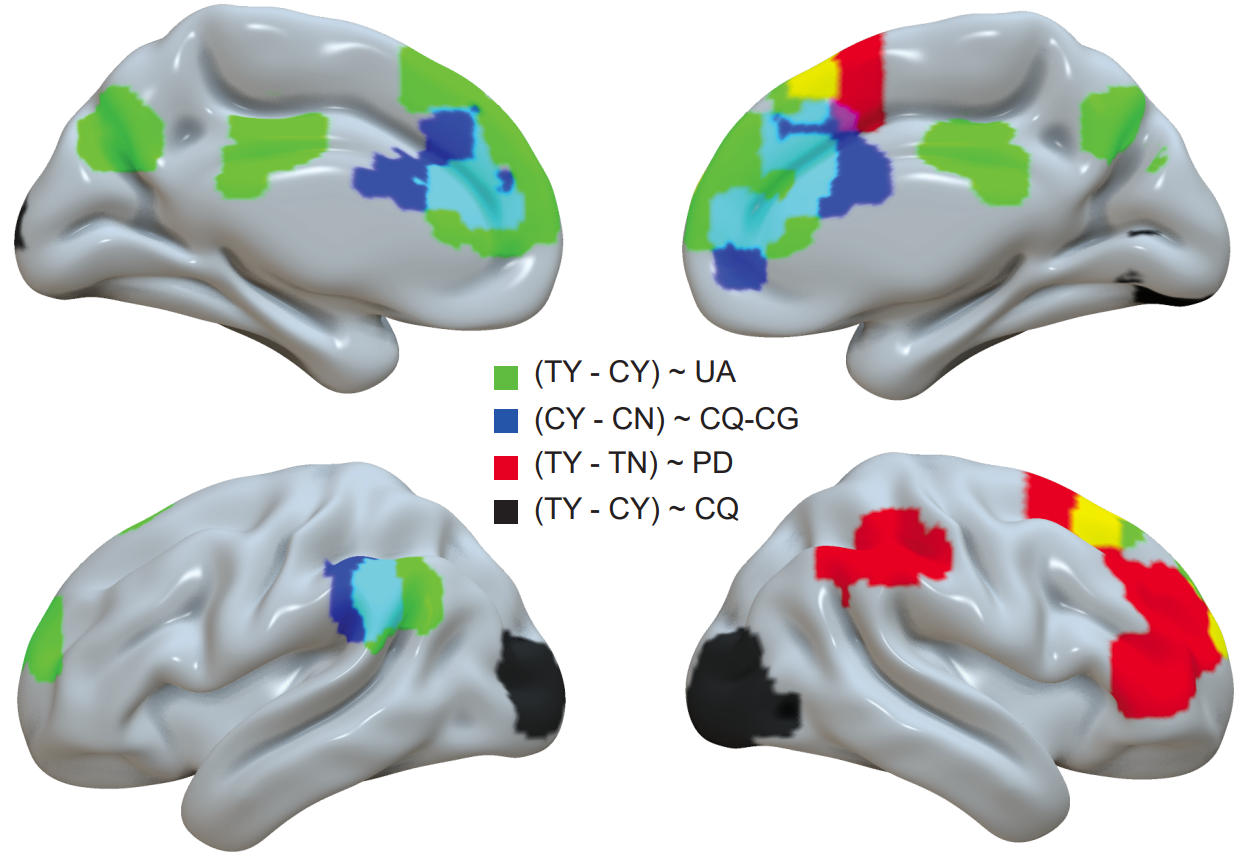


Fig. S6. Statistical map overlay simultaneously showing brain areas in which social preference questionnaire scores (UA, PD, CQ and CQ-CG) were significantly associated with accept vs. reject neural response contrast estimates for Teacher and Classmate conditions. Overlapping areas (cyan and yellow areas) includes anterior cingulate gyrus (cyan, 37 voxels), left inferior parietal gyrus (cyan, 3 voxels) and right superior frontal gyrus (yellow, 14 voxels). See also Table 2.
